# Step-by-step tutorial for analysis of Multiple Reaction Monitoring Profiling of lipidome data for biological interpretation

**DOI:** 10.7171/001c.163455

**Published:** 2026-06-30

**Authors:** Kristen Yamate, Linda Beckett, Theresa Casey, Christina R Ferreira

**Affiliations:** 1 Animal Sciences Purdue University West Lafayette; 2 Bindley Bioscience Center Purdue University West Lafayette

**Keywords:** Data analysis, MRM profiling, Data standartization

## Abstract

Multiple Reaction Monitoring (MRM) profiling analysis of lipids provides a sensitive semi-targeted approach to analyze the lipidome of biological samples. The aim of this manuscript is to describe the step-by-step analysis of MRM profiling data following acquisition on a triple quadrupole, with validation of the method demonstrated through the analysis of temporal changes in neonatal piglet liver lipidome between birth and 24 h postnatal. Following extraction of samples using the Bligh and Dyer method, lipids are profiled for a list of MRM scans on a triple quadrupole mass spectrometer. Data standardization is required to reduce technical variability, with the selection of the approach being guided by the biological question, sample type, or system being studied. Four approaches of data standardization are described requiring either pre-acquisition standardization or post-acquisition standardization: 1) raw intensity (pre-acquisition standardization only), 2) application of internal standard (requires both pre-acquisition and post-acquisition standardization), 3) relative abundance (percent) of a lipid relative to others within a class (post-acquisition standardization), or 4) sample injection per MRM list (post-acquisition standardization). After standardization, data are normalized for parametric statistical tests to explore hypothesis, with the use of Metaboanalyst 6.0 tools demonstrated as an option for user-friendly interface in the application of multivariate and univariate analyses. Biological interpretation of findings then proceeds through the use of pathway analysis and lipid ontology tools, such as Lipid Ontology (LION). Additionally, the analysis of differential lipids’ characteristics by carbon length and number of unsaturated bonds within lipid class is demonstrated. The workflow presented provides a practical framework for transforming raw MRM profiling data into biological insights. Standardization method shapes both the statistical outcomes and biological interpretation, emphasizing the importance of selecting an approach that is aligned with the biological question being asked.

## Introduction

Lipids are essential molecules that play an important role in biological systems, and lipidomics allow researchers to identify and profile thousands of lipids simultaneously using mass spectrometry (MS). Lipidomics analysis is achieved through several approaches, including multiple reaction monitoring (MRM) profiling, untargeted liquid chromatography tandem MS (LC-MS/MS), and desorption electrospray ionization-MS imaging (DESI-MSI).^1^ Within these approaches, MRM profiling uses the MS/MS scan that provides the best sensitivity to profile small molecules within a biological sample.^2^ A single MRM monitors a transition of a selected ion (precursor ion), which fragments into a corresponding smaller ion (product ion).^3^ Each precursor/product ion pair is related to the molecular structure of a lipid or metabolite, allowing for sensitive detection of small molecules. For example, the transition of *m/z* 760.6 to *m/z* 184.1 is related to the sum composition lipid nomenclature for phosphatidylcholine (PC) 34:1, which is a phosphatidylcholine lipid identified by the 184.1 product ion with 34 total carbons and 1 unsaturated bond across the 2 fatty acyl groups.

MRM profiling may be used as a semitargeted approach to lipidome analysis, as it relies on a predefined lipid database to determine the number and type of lipids interrogated in the samples. The MRM profiling workflow typically is run in two steps: a discovery phase and screening phase. The discovery phase aims to identify potential lipids of interest detectable in samples by scanning a large number of MRM in samples pooled by treatment or condition (e.g., health or disease) and comparing the data to a blank extract or just the solvent.^4^ The MRMs corresponding to lipids identified as being present in the samples during the discovery phase of profiling are then assembled into a list (often referred to as an “instrument method” containing a list of several MS scans) to profile individual samples only for MRM detected during the screening phase. We previously described the step-by-step approach for building the MRM profiling methods from the LIPID database and assembly of this list for the discovery phase and screening phase scans of the MRM-profiling method.^5^

Data acquisition from the screening phase produces a dataset of MRMs and related ion intensities, which is first standardized and then transformed for normal distribution prior to downstream statistical analysis and biological interpretation. We use the term standardization to describe preacquisition and postacquisition correction procedures that adjust raw lipid intensities relative to either an IS or the total signal within a biologically defined set.^6^ Preacquisition standardization encompasses measures taken to equalize sample inputs such as concentration, cell number, or starting material weight or volume, prior to data collection. Postacquisition standardization refers to the curative corrections applied after data are collected by adjusting lipid intensities relative to an IS or the total signal.^7^ These corrections minimize technical variation, ensuring that differences in signal intensity reflect biological rather than analytical variability. Following standardization, the data typically exhibit a skewed distribution and therefore require normalization to transform the standardized values toward a normal distribution suitable for parametric statistical analysis.^8^

Despite the growing use of MRM profiling for lipidomics, there is limited guidance on how to standardize and analyze MRM data for biological interpretation. The overall aim of this manuscript is to provide a step-by-step approach for data analysis following acquisition of MRM profiling scans. The step-by-step approach described here outlines three stages of MRM profiling postacquisition data analysis: standardization, statistical analysis (which includes normalization), and biological interpretation. This workflow is not intended to describe a single path, but rather it provides a structured framework that researchers can adapt to fit their biological question and available tools. The validation section of the manuscript describes the comparison of results of the four different standardization approaches that can be applied: raw intensity (no postacquisition standardization), IS within lipid class, sum of ion intensities within a lipid class, and sum of ion intensities within an MRM list profiled per sample injection. Results from this validation aim to help guide the selection of a standardization approach appropriate to the biological question being asked.

## Materials and Methods

### Data Acquisition and Output File Description

Previously, we described the approach to develop a list of MRM scans obtained from the Lipid Maps Standard Database (LMSD) corresponding to ~5,000 lipid species for semitargeted data acquisition on a triple quadrupole in the discovery phase.^5,9^ MRMs detected in the samples that are used in the discovery phase are then organized into lists (saved as instrument methods and referred to as “M”) for the screening phase of individual samples. The two-step method is designed to drastically reduce instrument usage time and noise when interrogating individual samples, as only MRMs detected in the pooled samples profiled during the discovery phase are profiled in individual samples during the screening phase.

The output data of MRM profiling are compiled into a list of the MRMs used for the screening phase and their respective ion intensities in each sample. As previously described, the list of MRMs profiled during the screening phase is usually split into different MRM lists, commonly referred to as “instrument methods” with numbers such as M1, M2, etc.^5^ Since several hundred MRMs are commonly selected to screen, the easiest way to obtain the list of ion intensities for each sample is to convert the proprietary file (“.d” in the case of Agilent Technologies) to mzML format, which is a standardized, open-source XML-based file format used to store and exchange MS data, including spectra, chromatograms, and metadata. Then, the mzML is parsed using scripts to generate.csv tables containing the lipids related to each MRM and the ion intensities recorded by the mass spectrometer for each of the samples. These tables serve as the output files that begin the step-by-step analysis of data described henceforth.

**Figure 1.**
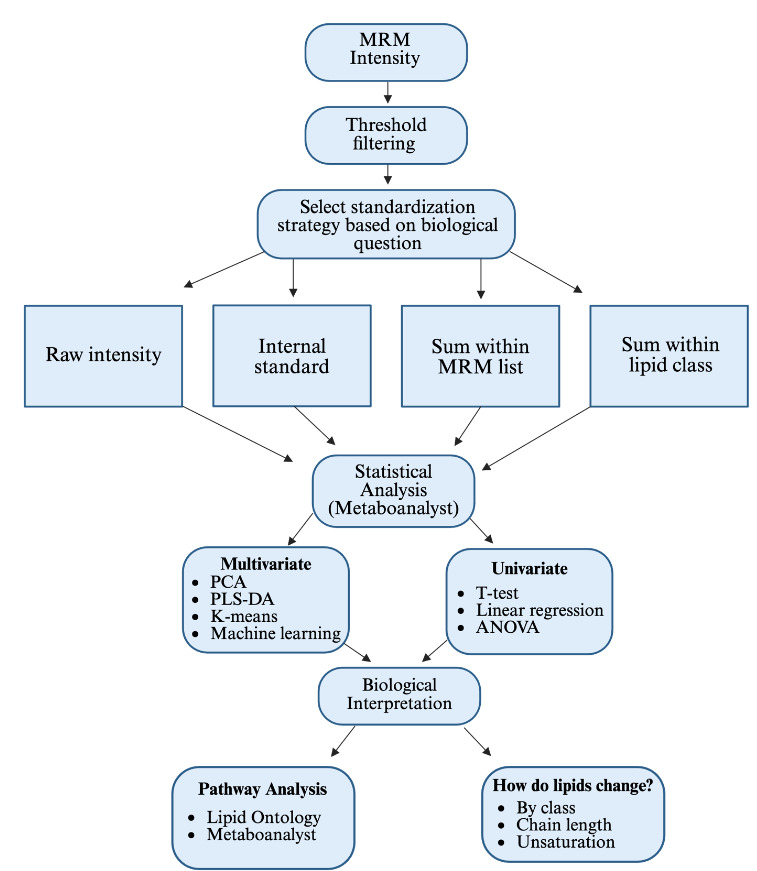
Biological Interpretation of Multiple Reaction Monitoring (MRM) Profiling Workflow. MRM intensities are threshold filtered and a standardization strategy is selected (raw intensity, IS, sum within MRM list per sample injection, or sum within lipid class). Standardized data can be analyzed with univariate and multivariate statistical approaches (e.g., using Metaboanalyst 6.0 or similar software). Biological interpretation is performed through pathway analysis and evaluation of lipid structural features (class, chain length, and unsaturation).

A structured workflow was developed to illustrate the steps required from postacquisition analysis and interpretation of MRM profiling data (Figure 1). The flow chart outlines the process, starting from the acquired MRM ion intensity data. The first step is to apply a user-defined threshold-based filtering to remove background signals (e.g., 1.5-fold higher than the blank). Based on the biological question of interest, users may choose one of the four standardization strategies: raw intensity (no postacquisition standardization), IS, sum within a lipid class, and sum within an MRM list profiled per sample injection. Once a standardization method has been applied, the dataset proceeds to the statistical analysis set. Following statistical analysis, results can be interpreted in a biological context by identifying lipid structural characteristics or by utilizing a pathway analysis software, such as lipid ontology (LION) or enrichment analysis tools in Metaboanalyst 6.0.

### Data Filtering

Filtering of the postacquisition MRM intensity data is the first step. In this step, lipids are selected for downstream analysis based on an intensity level greater than the blank (water). For example, for the validation study described as follows, MRM with intensities 1.5-fold above the blank (water) in at least one sample were selected. The 1.5-fold is arbitrary, is commonly used in metabolomics studies, and can be changed according to the user-based selected stringency, data analysis needs, or the objectives of the study. In the following workflow for data filtering, we include Excel commands and shortcuts to further help the user.

**Figure 2.**
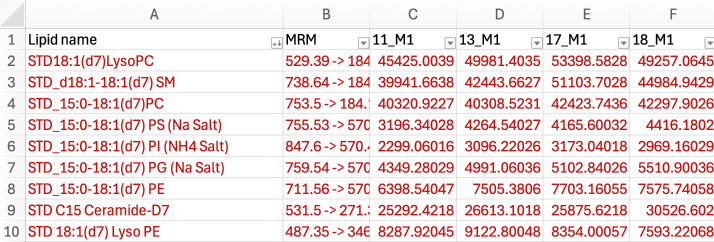
Identification of Internal Standards in the Working Dataset.

**Figure 3.**
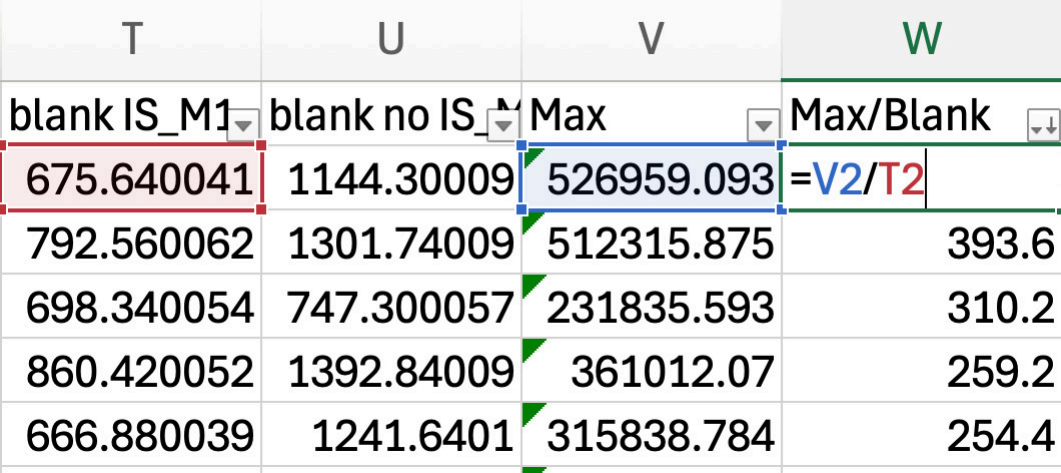
Example of Calculating Max/Blank Ratio for Determining Cut-Off Threshold in Excel.

1. After screening data acquisition, save all output folders (usually one folder for each list of MRMs). Open the first output file folder and make a copy of the absolute intensity Excel worksheet as a .xlsx file. Rename this file with the method (list of MRM profiled) and type of standardization applied (e.g., “Absolute_Intensity_M1_IS” or “Absolute_Intensity_M1_SUM”).
2. Name the first worksheet of the new Excel file as “Master”; this will serve as your master sheet where you will not alter the data.
3. Make a copy of the master sheet, create a new worksheet, and name it “Working”. The working sheet is used to edit the data, whereas the master worksheet ensures fidelity of the data is maintained.
  - You should now have two worksheets: a master and a working one.
4. If your dataset has ISs, highlight the entire working sheet and filter the lipid name by descending order. All the internal standards often appear at the top of the columns within the worksheet (depending on the letter of their naming, we used STD to indicate standards). Color all ISs red to easily identify them (see Figure 2).
5. The first step is to apply a filter to reduce background noise by removing MRMs that do not meet user applied cut-off of intensity values relative to blank sample intensity.
  - Find the MRM maximum intensity across all samples. Name the empty column next to the last column of data (e.g., it is blank intensity in Figure 3), “Max”. Use the equation =MAX (select the range of cells in the row corresponding to samples).
6. Next, determine a cut-off threshold. Name the column next to the Max “Max/Blank” and divide the Max intensity value by the blank intensity for each MRM corresponding to lipid species (see Figure 3). In our validation study, we chose to select the lipids for downstream analysis that were 1.5-fold above the blank.
  - If you are standardizing by an IS signal, use the blank spiked with ISs. If you are standardizing using the other methods, use the blank without the IS.

7. Highlight entire sheet and sort max/blank by descending order.
8. Remove the lines for all the MRMs that have a Max/Blank below your threshold. If your standard falls below your threshold, do not remove them from your sheet as they have been spiked in the blank and therefore will show low Max/Blank ratios.
9. Repeat steps 1-7 for all data method folders.

### Standardization by Raw Intensity

After filtering data to remove background noise as previously noted, no further processing steps are applied. Copy and paste all values from each method folder into one Excel worksheet and save as a .csv. The raw intensity values are used directly for downstream statistical analysis for this method.

### Standardization by Internal Standard (IS)

Standardization by IS requires an IS to be added to each sample at a known concentration. The IS corrects for technical variability during sample analysis. For IS standardization, each sample’s starting amount of material must be accurately known to ensure consistent IS addition. Standardization is performed by dividing each sample’s raw intensity by the IS’s intensity within the same sample of that lipid class. A video demonstration of standardization by IS workflow is showcased in the bundle within the PURR supporting documents.^9^ What follows is a step-by-step written protocol for standardization by IS, including Excel commands and shortcuts to further help the user.

**Figure 4.**
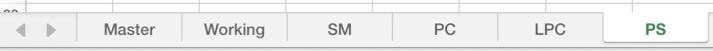
Example of Lipid Class Worksheets in Excel.

**Figure 5.**
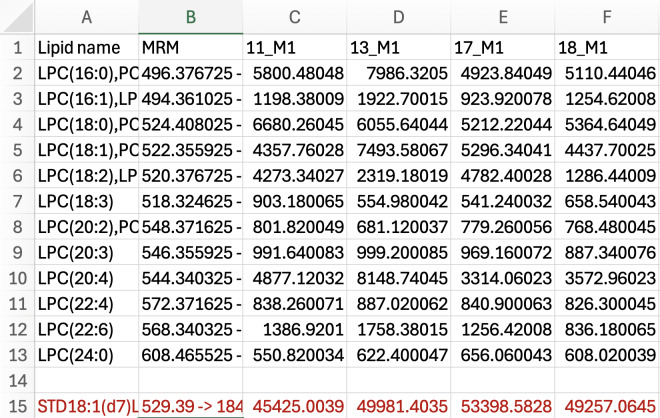
Example of Internal Standards Below Sample Intensity Data.

**Figure 6.**
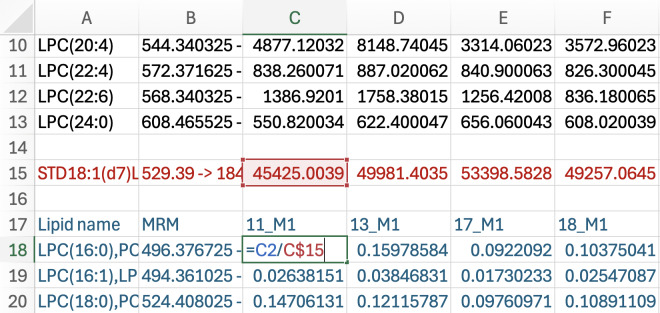
Example of Using a Formula to Standardize by Internal Standard in Excel.

1. After filtering data, identify rows with ISs (if following step-by-step sequentially, these will be in red font).
2. Make a worksheet for each lipid class that has an IS (see Figure 4).

3. For each worksheet corresponding to a lipid class, copy all the lipids in a class (e.g., lysophosphatidylcholine or LPC) with an IS from the “working” worksheet and paste into its own sheet. Copy and paste the standard intensities measured below sample intensity data (see Figure 5).

4. Make a copy of all the lipid intensities within the class and then paste it below the standard row. Color code this to blue to indicate that these will be standardized values.
5. Standardization: Working with the blue font cells containing intensity measured in each sample by each lipid class (see Figure 6).
  - Click on the first sample intensity in blue, then enter an equation to divide the original intensity (above in black font) by the standard intensity (e.g., =C2/C15).Makesureyouputa between the letter and number in the denominator of the equation to keep the standard relative to the sample.
  - Drag the formula to generate data normalized by the IS across all samples and double click the green box at the bottom corner of the last cell to apply the formula down through all data.

6. Repeat steps 1-5 for all classes of lipids with an IS in each method folder.
7. Create a new Excel workbook; this will serve as your summary file of all standardized lipids. Copy and paste values of all standardized values from the blue cells into the new workbook. Save the file as a.csv.

### Standardization by Sum Within MRM List per Sample Injection

Standardization by sum within the MRM list (or “method”; here referred to as M1, method 1 and M2, method 2, etc.) refers to the organization of lipid classes profiled per injection and varies by the number of lipids being interrogated. In the dataset example used in this manuscript, lists were grouped as M1 and included acylcarnitine (AC), phosphatidylglycerol (PG), phosphatidylserine (PS), phosphatidylethanolamine (PE), phosphatidylinositol (PI), PC, ceramide (Cer), and sphingomyelin (SM); M2 included cholesteryl ester (CE) and diacylglycerol (DG); M3 included triacylglycerol (TG); and M4 included free fatty acid (FFA). Within each list, the intensity of the individual MRM within a sample was standardized by dividing the sum of the ion intensities of all the MRMs in the list. This means of postacquisition standardization yields a beta-distributed dataset with values limited between 0 to 1. A step-by-step written protocol for standardization by sum within the MRM list per sample injection, including Excel commands and shortcuts to further help the user, is provided as follows.

**Figure 7.**
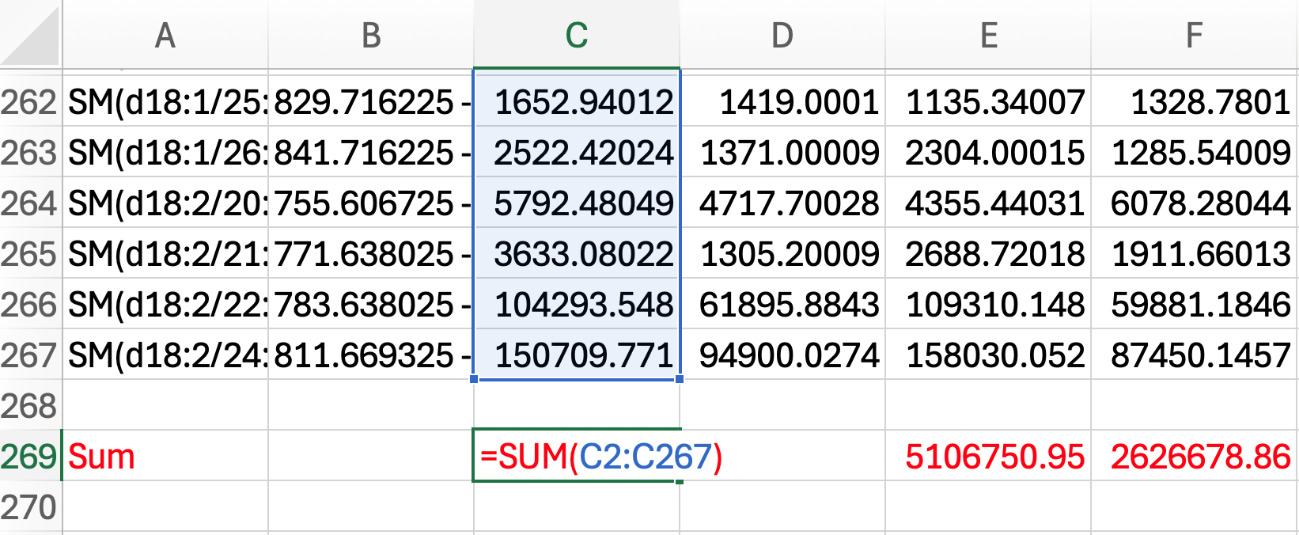
Example of Calculating the Sum of All MRM Profiling Intensities for One Sample in Excel.

**Figure 8.**
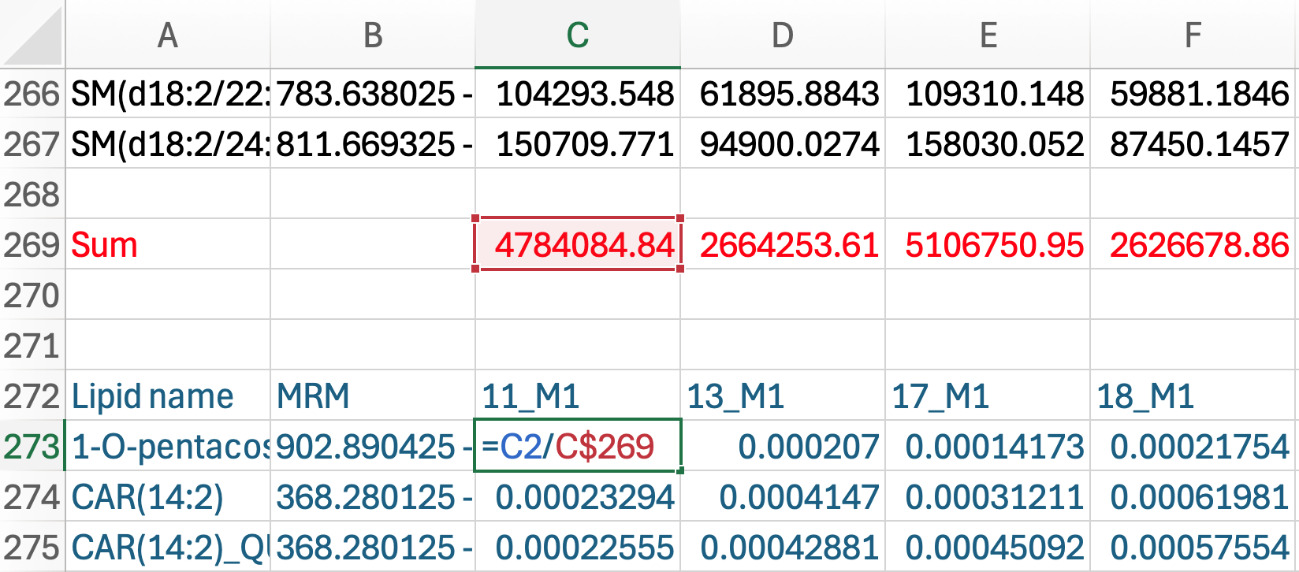
Example of Applying a Formula in Excel to Standardize by Sum Within MRM Profiling List and Within Lipid Class.

1. Choose a method (or MRM list) output file.
2. In the work sheet, name a row below the intensities “SUM” and color font red. Calculate the sum of all the MRM intensities for one sample (e.g., =SUM(C2:C267)), and then apply the formula across all samples (see Figure 7).

3. Make a copy of all the intensities of MRMs within the MRM list and paste it below the sum row. Color code this data in blue font to track these data as standardized.
4. For standardization and working with the blue font cells:
  - Click on the first sample intensity in blue, then put an equation to divide the original intensity (in black font) by the sum of that sample (e.g., =C2/C269).Makesureyouputa between the letter and number in the denominator of the equation to keep the sum relative to the sample (see Figure 8).

5. Drag formula across all samples and double click the green box at the bottom corner of the last cell to apply the formula down.
6. Repeat steps 1-5 for the other instrument methods’ output files.
7. Create a new Excel workbook; this will serve as your summary file of all standardized lipids. Copy and paste values of all standardized values from the blue cells into the new workbook. Save the file as a .csv.

### Standardization by Sum Within a Lipid Class

To standardize by sum within a lipid class, MRMs were separated by the class attribution (AC, SM, PG, Cer, PS, PE, PI, PC, CE, DG, TG, and FFA). The intensity of all MRM within a class are summed for each sample, and then intensity values for each MRM within the sample are divided by the sum to yield a value equal to the proportion (percent) of that lipid relative to other lipids in a class in the sample. This means of postacquisition standardization yields a beta-distributed dataset with values limited between 0 to 1. What follows is a step-by-step written protocol for standardization by sum within a lipid class, including Excel commands and shortcuts to further help the user.

1. Highlight the entire working sheet and sort by lipid name. Each lipid class should be grouped together.
2. Make a sheet for each lipid class. Copy and paste all the intensities for each class to their respective sheet (see Figure 4).
3. In the lipid class sheet, name a row below the intensities “Sum” and change the color to red. Take the sum of all the lipid intensities for one sample (e.g., =SUM(C2:C4)), then drag the green box at the bottom corner of the red cell across all samples.
4. Make a copy of all the intensities of MRMs within the class and paste it below the sum row. Color code this data using blue font.
5. For standardization and working with the blue font cells,
  - Click on the first sample intensity in blue, then put an equation to divide the original intensity in black by the sum of that sample (e.g., =C2/C6).Makesureyouputa between the letter and number in the denominator of the equation to keep the sum relative to the sample (see Figure 8).
  - Drag the formula across all samples and double click the green box at the bottom corner of the last cell to apply the formula down.
6. Repeat steps 1-5 for all lipid classes in each method folder.
7. Create a new Excel workbook; this will serve as your summary file of all standardized lipids. Copy and paste values of all standardized values from the blue cells into the new workbook. Save the file as a .csv.

### Lipid Ontology (LION)

LION is a pathway analysis tool that can help guide biological interpretation of your standardized MRM datasets.^10^ LION classifies lipids in a dataset to physical or chemical properties, function, and cellular components. The dataset standardized by sum within a lipid class was analyzed in ranking mode, where local statistics from a one-tailed *t*-test were applied to rank lipid identifiers between conditions. Enrichment was evaluated using a Kolmogorov-Smirnov test with the ranking direction set from low to high and the alternative hypothesis specified as one-tailed. The following step-by-step list explains how to prepare the data file for LION analysis, including Excel commands and shortcuts to further help the user.

1. After standardizing screening data by one of the previously described four strategies, edit the file to a format readable by LION software and by comparisons of interest. For example, what lipids significantly increased or decreased in response to treatment. Output data often have multiple species of isobaric lipids corresponding to a single MRM (for example, PG 34:0, PG O-35:0). Since these lipid isobars cannot be distinguished, the likelihood of each species needs to be given similar weight, and so they need to be entered individually as a row of data. To prepare your input file:
  - Remove the MRM transition column, as LION cannot read this information.
  - Insert six columns between the lipid name and the first sample column. This provides enough empty space for Excel to separate lipid species.
  - In the lipid name column, replace the semicolon with a comma by using the command Find & Replace all “;” to “,”.
  - Convert text to column using the comma as the delimiter. This separates the isobaric lipid species that were listed together in a single cell into individual entries.
  - Copy and paste each separated lipid into new rows.
    i. It is helpful to color the text for the copied values to ensure you are not missing values (no need to delete or combine duplicates, LION combines this for you).
    ii. Ensure that lipid name and intensities are together.
  - Once each lipid has its own line, remove the following using the command Find & Replace (replace with nothing):
    i. Brackets
    ii. Neutral loss (NL)
    iii. Adducts from CE (Na, H, K)
  - Organization of the file should be:
    i. Row 1: Treatment
    ii. Row 2: Sample ID
    iii. A1: Lipid Name
    iv. A2: Blank
  - Save file as a .csv.
2. Then upload the file into LION using the following steps.
  - Ranking mode > file input > Choose the .csv file.
  - Select local statistics to rank input identifiers: one-tailed *t*-test (2 conditions).
  - Condition of interest (test): for example, stay on sow (SOS); Control condition (comparator): for example, zero hour (ZH).
    i. The correct comparison depends on the biological question.
  - Click calculate local statistics > use values as local statistics.
  - Kolmogorov-Smirnov settings.
    i. Ranking direction: low to high.
    ii. Alternative hypothesis: one-tailed.
  - Submit.
  - At the top there are tabs for the following.
    i. General information.
    ii. LION input: matches our library to the LION library.
    iii. LION enrichment table: annotated, *p*-value, and FDR *q*-value.
    iv. LION enrichment graph: ranks significant groups.
    v. LION network view: lipid classification, physical or chemical properties, function, and cellular component.

### Statistical Analysis

The standardized dataset can be uploaded to Metaboanalyst 6.0 for normalization and statistical analysis.^11^ Metaboanalyst 6.0 is a commonly used statistical platform due to its user-friendly interface and built-in tools for univariate and multivariate analysis. Other statistical programs are available, but we emphasize Metaboanalyst 6.0 here because it is freely available and does not require the user to have to code. Data normalization approach should be driven by the dataset and knowledge of the user; typically for the autoscaling of our datasets, that normalization centers the data by dividing the mean by the standard deviation of each variable. Once data are normalized, Metaboanalyst 6.0 tools can be used for multivariate and univariate analysis. Our validation studies applied principal component analysis (PCA), volcano plots, K-means clustering, and hierarchical cluster analysis using data standardization by sum within the lipid class, as well as *t*-test analysis with correction.

### Lipid Characteristic Analysis

Following statistical analysis to identify lipids impacted by treatment or time, their species characteristics can be analyzed to help guide biological interpretation. TG and DG that are different can be characterized by chain length, unsaturated bond number, and fatty acids identified by NL product ions. For example, TG54:4_NL 18:3 refers to a TG with 54 total carbons and 4 unsaturated bonds across the 3 fatty acyl groups, and the 18:3 reflects the fatty acyl group profiled by its NL in the ion transition. To determine how lipid structural features differed between treatments in our validation experiment described as follows, *t*-test results were generated in Metaboanalyst 6.0 using a significance threshold of FDR <0.05. Lipid species that do not meet this threshold were excluded from downstream analysis. Significant lipids were sorted by *t*-statistic to identify species that increased or decreased with treatment. Positive *t*-values were classified as increased in the comparator, whereas negative *t*-values were classified as decreased in the comparator. TG names were separated using text-to-column function in Excel to isolate structural features including carbon number, unsaturation, and NL. For each group (increased and decreased with corresponding structural feature), the total number of TGs within the group was summed to serve as the denominator. The percent of each feature was calculated as the number of TGs possessing that specific carbon length, unsaturation, or NL divided by the total number of TGs in the group. Proportional values were then compiled into summary tables and visualized as bar graphs to compare the distribution of TG characteristics between treatments. Although we demonstrate this analysis using the TG class, the approach can be extended to other lipid classes such as DGs and membrane lipids to examine class-specific compositional shifts; however, NL product ions are attributes only related to DG and TG MRM profiles.^12^ The following steps explain how to interpret lipid characteristics including Excel commands and shortcuts to further help the user.

**Figure 9.**
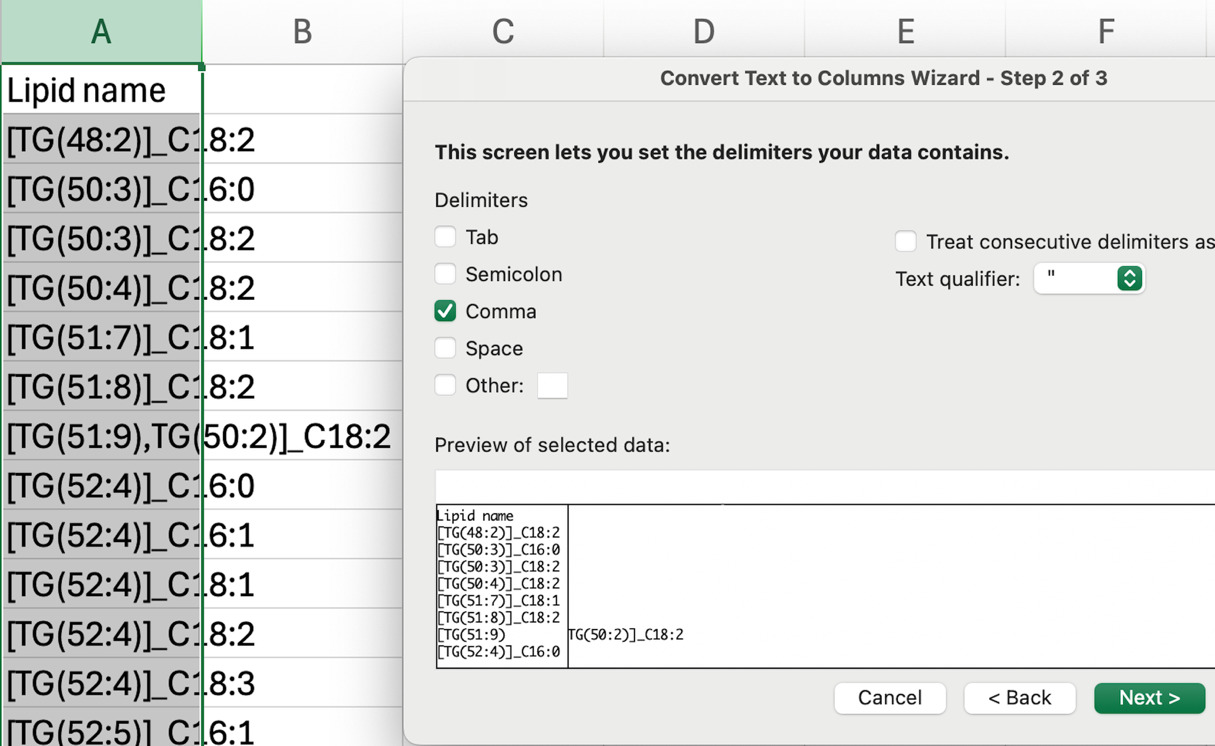
Example using Convert Text to Columns Wizard Function in Excel to Separate Lipid Characteristics.

**Figure 10.**
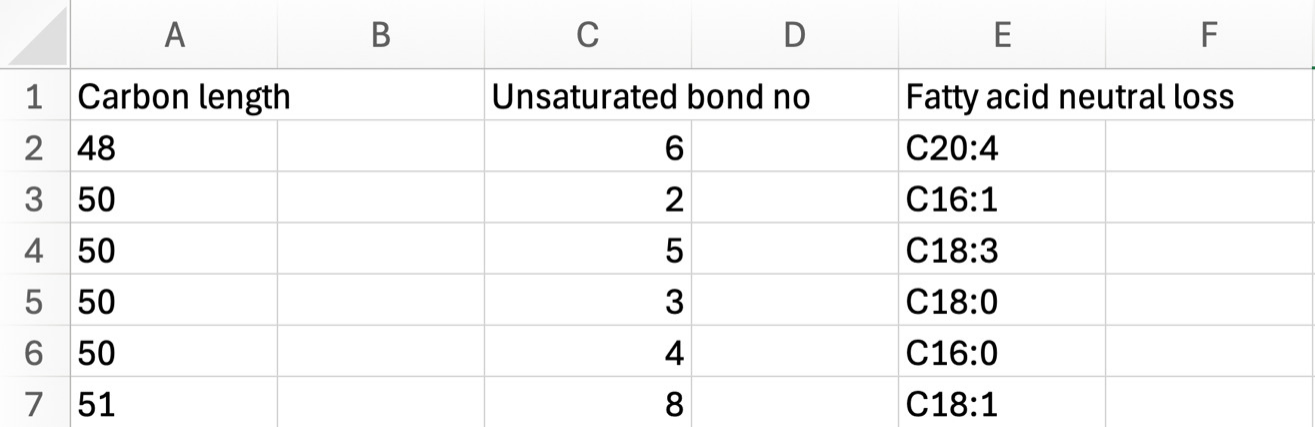
Example of the Excel File After Extracting Carbon Length, Unsaturated Bond Number, and Fatty Acid NL Characteristics from Lipid Name.

**Figure 11.**
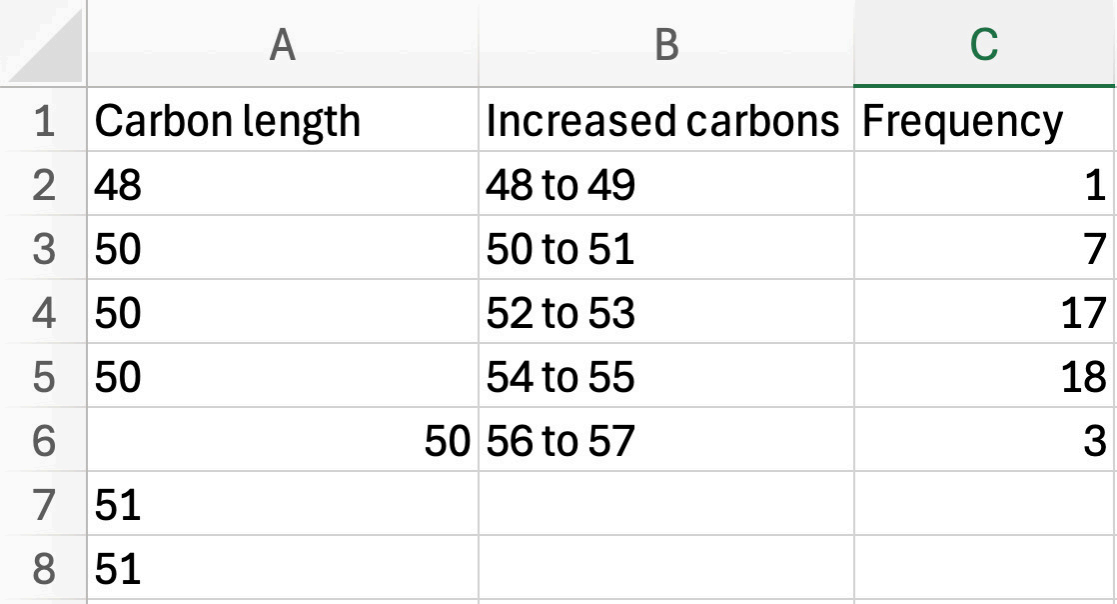
Example of Identifying Frequency of Even-Odd Carbon Chain Lengths.

**Figure 12.**
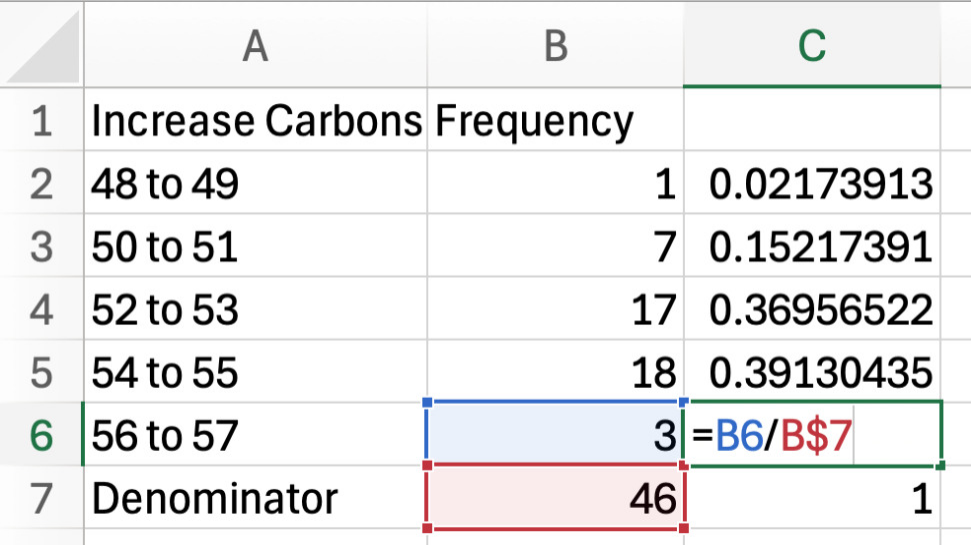
Example of Calculating the Frequency Proportion for Carbon Chain Length in Excel.

**Figure 13.**
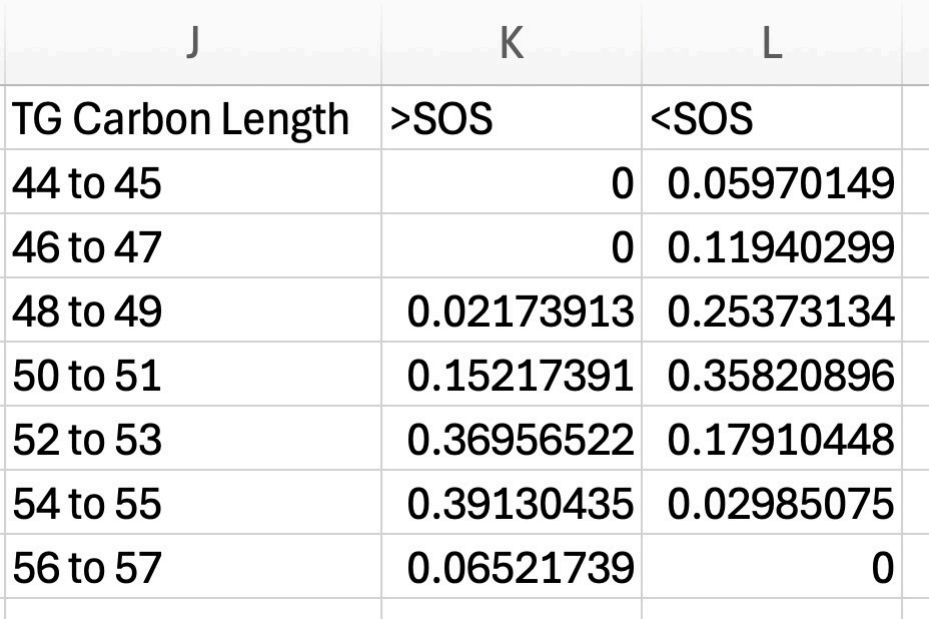
Example of Summary Table of the Carbon Length Proportion for Both the Increased (>SOS) and Decreased (<SOS) TG Group.

**Figure 14.**
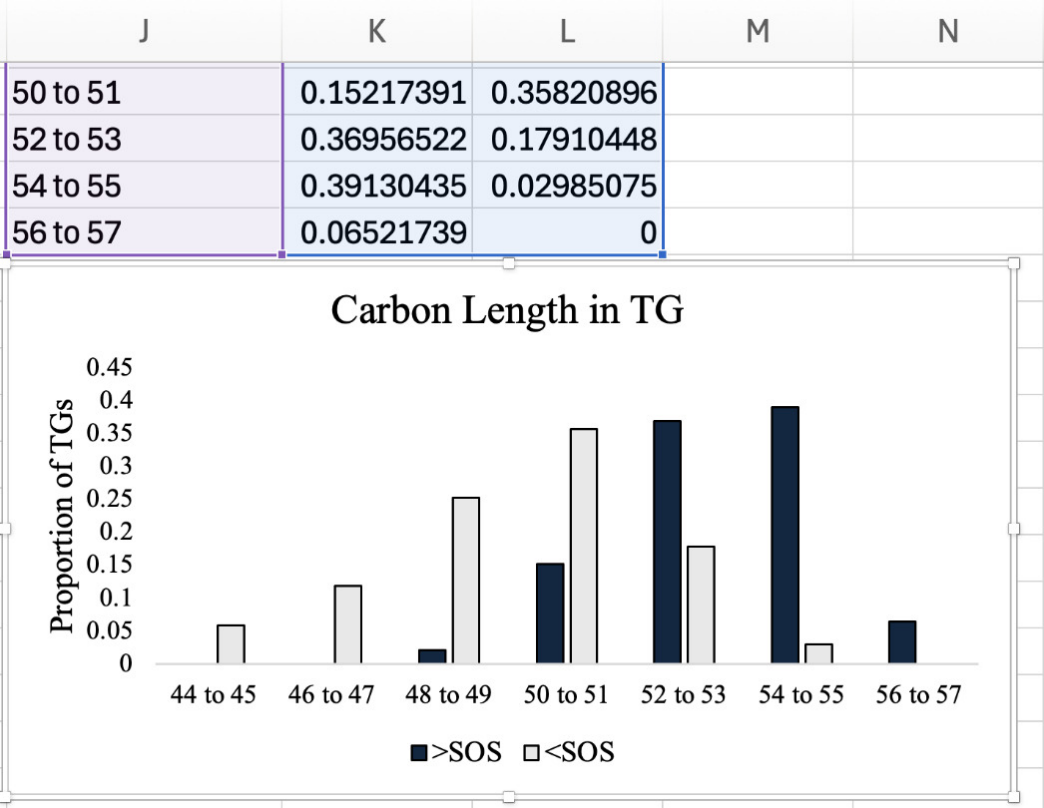
Example of Graphing the Summary Table in Excel.

1. Prepare Excel workbook.
  - After obtaining *t*-test results, rename the first sheet “Master”; this will serve as your master sheet where you will not touch the data.
  - Make a copy of the master sheet and name the new sheet “Working”. The working sheet is used to sort data by your statistical cut-off (*p*-value or FDR).
    i. For the validation dataset, we chose an FDR <0.05 as our threshold.
  - Create a new sheet titled “Sorting”, then copy and paste all the values that meet the significance threshold.
    i. Highlight the entire sorting sheet and sort by lipid name in ascending order. All the TGs should be grouped together.
  - Create a new sheet named “TG”, then copy and paste all TGs into this sheet.
    i. The Metaboanalyst 6.0 *t*-test output includes a column labeled t.stat containing the *t*-values. Highlight the entire TG sheet and sort by t.stat column in ascending order.
    ii. Positive *t*-values = higher with treatment.
    iii. Negative *t*-values = lower with treatment.
2. Extract the lipid characteristics from lipid name (carbon length, unsaturated bond number, and fatty acid NL).
  - Create a new sheet named “TG increased”, then copy and paste all the positive *t*-values from step 4 into this sheet. Keep column A as the lipid name. Delete the other columns.
  - Separate TGs that contain more than one lipid, for example [TG 55:10, TG 54:3]_C16:0).
    i. Highlight column A, and convert text to column using comma “,” as the delimiter to separate multiple lipids. Then, copy and paste the separated lipid entries into a single column (column A) (see Figure 9).
  - Separate fatty acid NL.
    i. Highlight column A and convert text to column using underscore “_” as the delimiter. Cut the fatty acid NL column and paste into a new column (e.g., column I).
  - Separate the unsaturated bond number.
    i. Highlight column A and convert text to column using colon “:” as the delimiter. Cut the unsaturated bond number column and paste into a new column (e.g., column E).
  - Clean unsaturated bond number column.
    i. Highlight the column (containing the unsaturated bond number). Using Find & Replace, find parentheses and brackets and replace them all with nothing. You want to make sure you only have numbers in this column.
  - Separate carbon chain length.
    i. Highlight column A and convert text to column using open parenthesis “(” as the delimiter. Name the column “column carbon length.”
  - Delete the column that contains “[TG”. Sheet should have columns dedicated to carbon length, unsaturated bond number, and fatty acid NL extracted from the lipid name (see Figure 10).
    i. Tip: Add blank columns between characteristics so you can sort each one independently without disrupting the rest of the data. This will be useful in the next steps.

3. Carbon length: sort the carbon length column by ascending order.
  - In column B, group the carbon lengths into even-odd chain length ranges (e.g., 48–49, 50–51, etc.) based on the values present in your dataset.
  - In column C, count how many lipids fall within each carbon length range and enter that value as the frequency (see Figure 11).

4. Unsaturated bond number: sort the unsaturated bond number column by ascending order.
  - In column G, count how many times each unsaturation number appears and record the count next to the first occurrence. Then, sort the column so the matching values are grouped together.
5. Fatty acid NL: sort the fatty acid NL column by ascending order
  - In column L, count how many times each fatty acid NL appears and record the count next to the first occurrence. Then, sort the column so the matching values are grouped together.
6. Repeat the “Extracting lipid characteristics from lipid name” steps 1–10 for negative *t*-values (TG lower with treatment).
7. Graphing lipid characteristics.
  - Create a new sheet named “TG carbon length graph”, then copy the carbon length ranges and their frequencies (for both the increased TGs and decreased TGs) into this new sheet so they are side-by-side.
  - Calculate the total frequency by using the SUM function (=SUM(B2:B6)) for both.
  - Next to the frequency column, calculate the proportion of the frequency for one carbon length by dividing its frequency by the total frequency (the denominator). Complete this for both increased and decreased carbons (see Figure 12).
  - Once you have calculated the proportion of each carbon length for both the increased and decreased TG groups, place them into a single side-by-side summary table (see Figure 13). Columns include:
    i. Combined carbon length ranges.
    ii. For our validation dataset: > SOS (higher in SOS).
    iii. For our validation dataset: < SOS (lower in SOS).
  - Highlight the summary table and insert a clustered column graph as shown in Figure 14.

8. Repeat “Graphing lipid characteristics” steps 1–4 for the other lipid characteristics.

### Method Validation

The samples analyzed were from a larger previously described study reviewed and approved by the Institutional Animal Care and Use Committee of Purdue University (Purdue IACUC Protocol No. 2110002200).^13^

Two groups of gilts (weighing 1.2–1.8 kg at birth) from the larger experiment served as sources of liver tissue samples used in the validation study described: ZH (n = 8), which were euthanized immediately after birth, and SOS (n = 9), which suckled from the dam for the first 24 hours postnatal for *ad libitum* intake of colostrum. Following the 24-hour suckling period, SOS pigs were fasted for 2 hours and then euthanized. Piglets were euthanized by CO_2_ inhalation administered by slow fill, an American Veterinary Medical Association approved method.^14^

**Figure 15.**
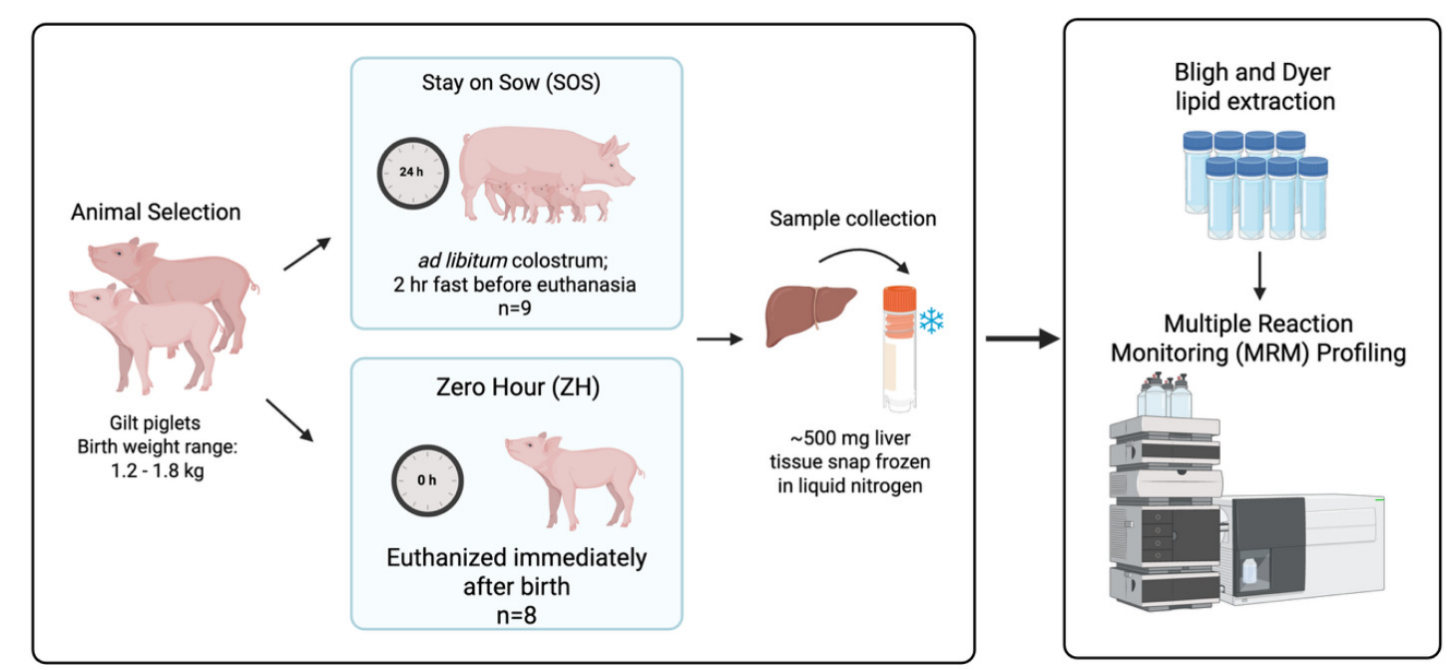
Experimental Design. Gilt piglets (birth weight 1.2–1.8 kg) were assigned to either SOS group, which stayed on the sow and suckled colostrum *ad libitum* for 24 hours followed by a 2-hour fast prior to euthanasia (n = 9), or the ZH group, which was euthanized shortly after birth (n = 8). Approximately 500 mg of liver tissue was collected, snap frozen in liquid nitrogen, and lipids were extracted using a Bligh and Dyer protocol for targeted multiple reaction monitoring (MRM) profiling.

Following exsanguination, the peritoneum was open, the liver was excised from the body cavity, and the right lateral lobe of liver was removed. Liver was sectioned into approximately 500-mg pieces of tissue, placed in a cryovial, and snap frozen in liquid nitrogen. Samples were temporarily stored in a -20ºC freezer on dry ice and then placed in a -80ºC freezer until extraction of lipids from samples (Figure 15).

For preacquisition standardization, liver samples were weighed prior to extraction using the Bligh and Dyer technique.^15^ Following recording of weight, liver samples were homogenized in ultrapure water at a ratio of 20 mg/100 µL using 2 mL tubes containing 1.4 mm ceramic (zirconium oxide) beads (Fisherbrand cat# 15340153, ThermoFisher Scientific, San Jose, CA), and 200 µL of this homogenate was used for the lipid extraction. After this, 550 µL of methanol (MeOH) and 250 µL of chloroform (CHCl_3_) were added to the liver homogenate and vortexed for 10 seconds to become a one-phase solution. Samples were incubated at 4˚C for 15 minutes. After incubation, 250 µL H_2_O and 250 µL CHCl_3_ were added and centrifuged at 15,000 x g for 5 minutes to generate a two-phase solution. From the nonpolar phase, 200 µL were transferred into 2 mL microcentrifuge tubes, and the solvent was evaporated using a Speedvac (Savant Speedvac AES2010, ThermoFisher Scientific) for 2 hours. The dried samples were stored at -80ºC until MRM profiling analysis.

MRM profiling data acquisition was performed using an Agilent 6410 Triple Quadrupole mass spectrometer (Agilent Technologies, Santa Clara, CA). Dried down lipid extracts were resuspended with 200 µL of MeOH, the same volume that was dried down, to generate a lipid extract stock solution. Each stock solution was then diluted 1:150 in the injection solvent [acetonitrile (ACN): MeOH: 300 mM ammonium acetate (NH_4_Ac) with ratios 3:6.65:0.35, respectively]. For the discovery phase, samples were pooled by treatment and 8 µL of each sample was introduced into the electrospray ionization source using a G1377A microautosampler. The precursor ion selection window was set to 0.7 Thomson, with the capillary pump delivering the solvent at 10 µL/minute under a gas pressure of 150 bar. Instrument conditions included a capillary voltage of 3.5 to 5 kV and a drying gas flow of 5.1 L/minute at 300ºC.

The list of MRM transitions used in the discovery phase was based on the methods described here.^5^ Since the previous publication, the Lipids Maps online database (http://www.lipidmaps.org/) characterized additional lipid species. Although we used the previous methods, an updated version of the MRM transition list including the newly identified lipids is available through the Purdue University Research Repository for future use.^9^ Following the discovery phase, MRM transitions with raw ion intensity values 1.3-fold higher than the blank extract (water) in the pooled samples were selected for the screening phase in individual samples, resulting in a total of 906 MRM transitions.

During the screening phase, individual samples were profiled using these 906 MRM transitions. Each sample for the screening phase was prepared using the same previously described steps and additionally spiked with EquiSplash LIPIDOMIX Quantitative Mass Spec Internal Standard (Avanti Research, Alabaster, AL, USA). The lipid classes with ISs were: PG, PS, PE, lysophosphatidylethanolamine (LPE), PI, PC, LPC, Cer, SM, CE, DG, and TG. AC and FFA did not have an IS. MRMs were organized into four acquisition lists, saved as instrument methods M1–M4. The number of MRMs for each list contained fewer than 500 MRMs due to a software limit regarding the number of transitions per method and only one ionization mode. All lipids were evaluated with the positive ion mode except for FFA, which were monitored using the negative ion mode. Accordingly, MRM lists were organized as: method 1 (M1) (which included MRMs related to AC, PG, PS, PE, PI, PC, Cer, and SM), method 2 (M2) (which included CE and DG), method 3 (M3) (which included TG), and method 4 (M4) (which included FFA). M1 grouped MRMs associated with membrane lipids together. M2 and M3 had only two or one lipid classes listed due to the large number of transitions for these classes. M4 MRMs were acquired in the negative ion mode using single ion monitoring because FFA do not present informative product ions by collision-induced dissociation without derivatization. The MRMs related to selected isotopically labeled lipids from the EquiSplash LIPIDOMIX were added to the M1, M2, and M3 to monitor the spiked IS signal.

### Workflow overview

**Figure 16.**
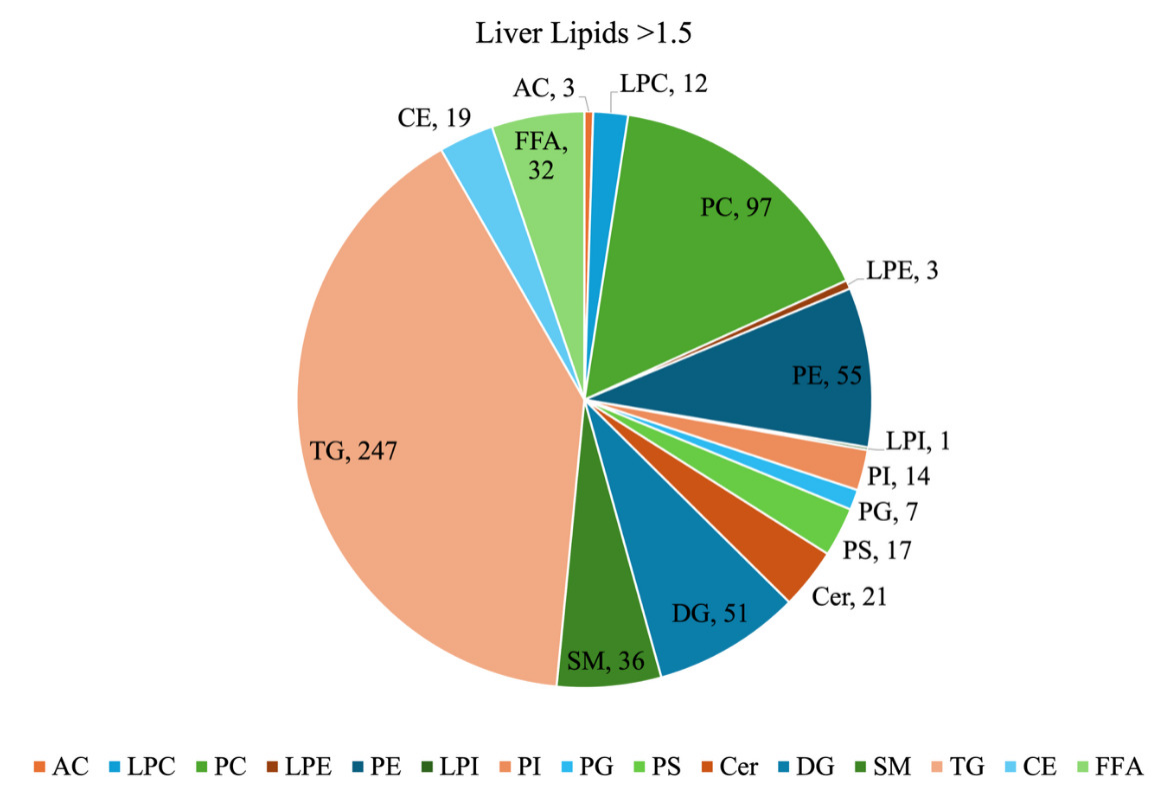
Liver Lipid Class Distribution. Pie chart showing the number of liver lipid species that exceeded the intensity threshold (1.5-fold above the blank) summarized by lipid class: AC, PG, PS, PE, PI, PC, Cer, SM, CE, DG, TG, and FFA.

For our sample dataset, a filtering threshold of 1.5-fold above the blank was applied to the screening step MRM intensity data to reduce background noise. After filtering, a total of 615 MRM were retained and classified by lipid class (Figure 16). TG were the most abundant class, representing 40% of the total number of lipids detected. Other relatively abundant classes were PC at 16%, PE at 9%, and DG at 8%. The class distribution provides a general overview of the neonatal piglet liver lipidome. Nonetheless, it should be noted that since the MRMs used for TG lipids have a product ion related to a fatty acyl chain NL, the same TG may be detected more than once due to its different fatty acyl chain composition.

### Standardization approach shapes biological interpretation

**Figure 17.**
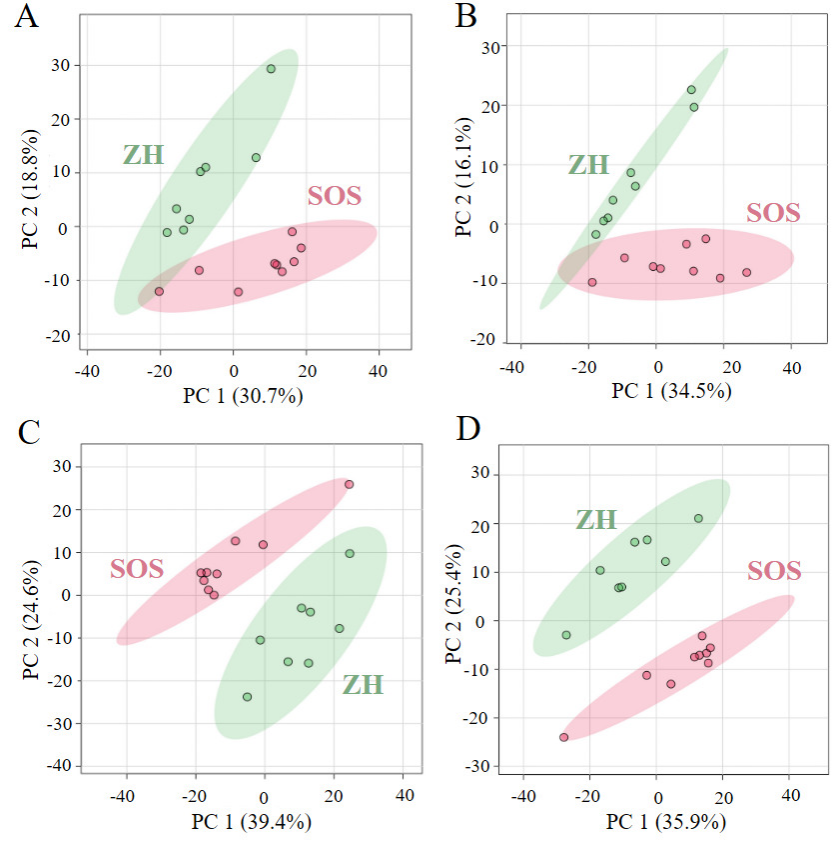
Principal Component Analysis (PCA) of Liver Lipid Profiles Using Different Standardization Strategies. PCA 2D scores plots are shown for (A) raw intensity, (B) IS, (C) sum within MRM list per sample injection, and (D) sum within lipid class standardization. Raw intensity and IS approaches are analogous to molar concentration, whereas sum within MRM list and sum within lipid class are analogous to molar proportion. Pink and green represent SOS and ZH, respectively.

Standardization strategies can substantially shape the interpretation of lipidomics data.^7,16,17^ We categorize the previously described standardization approaches as concentration-based and proportion-based approaches, roughly equivalent to molar concentration and molar proportion of lipids in a sample. The previously described concentration-based standardization approaches include raw intensity (no postacquisition standardization) and IS. The proportion-based approaches include standardization by sum within lipid class and standardization by sum within MRM list. Expressing data both as molar concentration and molar proportion is biologically relevant, but they capture different aspects of the system. As shown in the 2D scores plot of PCA, the degree of separation between SOS and ZH varied depending on the standardization method applied (Figure 17A–D). Molar concentration-based approaches exhibited a moderate separation. In contrast, molar proportion-based approaches produced more apparent separation and tighter clustering. These differences emphasize distinct aspects of the lipidome depending on whether concentration or proportion is prioritized.

Raw intensity, representing the total ion signal directly produced by the instrument, can be used as a starting point for statistical analysis and is occasionally used in untargeted MS studies. However, using raw intensity data adds technical variation, the specifics of which are beyond the scope of this manuscript but have been described by others.^18,19^ In general, although often used for downstream analysis, raw intensity data should be interpreted with caution. Metabolomics analysis of urine using ultra-high performance liquid chromatography-quadrupole time-of-flight mass spectrometry (UHPLC-QTOF-MS) found that use of raw intensity introduced variability driven by electrospray ionization contamination, and authors indicated that use of raw intensity should be limited to quantifying drift and concentration effects rather than for biological interpretation.^20^ When raw intensity is used, statistical platforms typically apply a log transformation to reduce skewness and normalize the distribution.^7^

IS standardization provides a more selective and accurate measure of lipid abundance by correcting the signals relative to a reference compound that behaves similarly to endogenous lipids.^21^ The use of ISs minimizes technical variability and improves the consistency of lipid signal measurements across samples. The caveat is that this approach requires accurate knowledge of the starting biological material to determine the appropriate amount of IS to add, making it impractical for applications where the initial material is unknown or highly variable, such as samples collected by swabs, saliva, seminal plasma, and sweat.^22^ In this workflow, our IS standardization was limited because our IS mixture did not include standards for AC and FFA, so these lipid classes were excluded from the IS standardized analyses. As a result, the pool of lipids considered for differential abundance contained 35 fewer MRMs (3 AC and 32 FFA) than the other standardization approaches. When starting material is known, IS standardization reflects molar concentration, which makes this approach most appropriate for questions that depend on absolute lipid levels, such as reaction rates, enzyme activity, or substrate availability in diets and digesta.

Standardization by sum within lipid class and by sum within MRM list both result in beta-distributed data, which approximates molar proportion of the lipid within the lipid class or MRM list, respectively. Both sum-based standardization methods become necessary when the amount of starting biological material is unknown. Since these standardization approaches express lipid values as proportions that must sum to one, an increase in an individual lipid will lower the calculated proportions of others, even if their absolute abundance does not change. Standardization by sum within the MRM list assumes that all lipid classes within that list scale proportionally, which can create bias if certain classes dominate over others.^23^ In our dataset, standardization by sum within MRM list identified lipid species different (FDR < 0.05) in the PE, CE, PS, PI, and SM classes than standardization by sum within lipid class. The class-based method of standardization would have required relaxing the FDR threshold to 0.98 to recover all lipids identified when standardization was done by the MRM list (instrument method, M). This suggests that the organization of transitions into MRM lists can shape which lipid classes are found significantly different and should be constructed with the expected biological lipid composition in mind. Standardization by sum within classes partially addresses this limitation by scaling lipids relative to the total intensity of their own class, thus preserving the relative proportions of lipids within that class.

**Figure 18.**
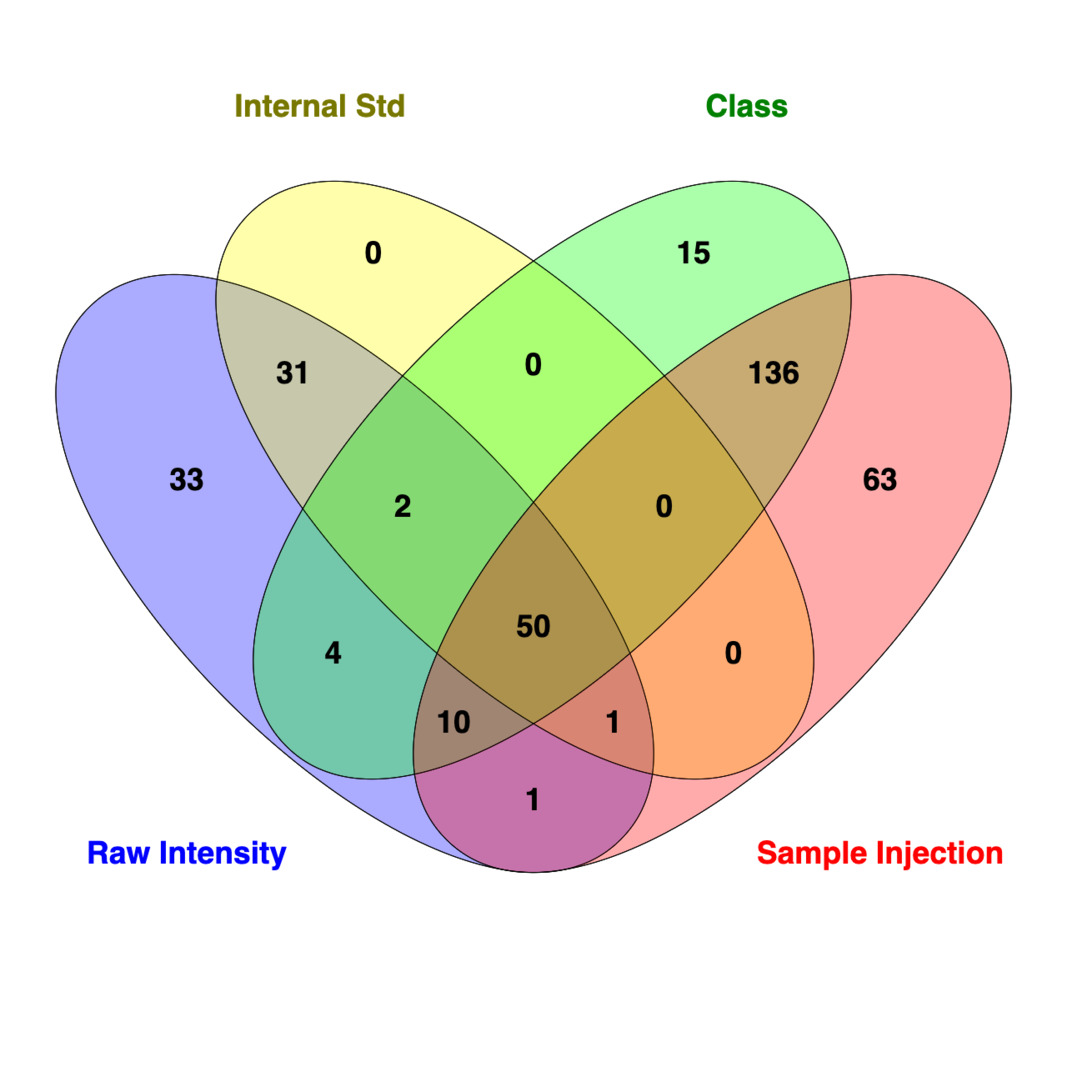
Standardization Strategy Detection Venn Diagram. Venn diagram showing the overlap in liver lipids that differed between SOS and ZH (FDR <0.05, *t*-test in Metaboanalyst 6.0) when data were standardized by raw intensity, IS, sum within MRM list profiled per sample injection (sample injection), and sum within lipid class (class). Of the 691 lipids tested, 50 were consistently identified as different between treatments across all four standardization approaches.

To further assess how standardization strategy affects interpretation of data, a Venn diagram was used to compare the number of lipids that are significantly different (FDR <0.05) between SOS and ZH identified using *t*-test analysis in Metaboanalyst 6.0 for each standardization approach (Figure 18). While 50 of the 691 lipids were consistently identified as different between treatments across all four methods, it should be noted that the IS approach evaluated a reduced lipid pool (n = 656), as AC and FFA were excluded due to a lack of corresponding ISs. Each standardization strategy revealed a different number of differentially abundant lipids with *t*-test analysis finding 132, 83, 261, and 217 for raw intensity, IS, sum within an MRM list profiled per sample injection, and sum within a class, respectively. For approaches using molar concentration standardization, all lipids identified as differentially abundant between groups standardized using IS overlapped with those identified with raw intensity. Compared to the IS approach, there were 48 additional lipids identified as different in the raw intensity data. Notably, only two lipids belonged to classes excluded from IS standardization (one AC and one FFA). For molar proportion standardization strategies, *t*-test analysis of data found 196 differentially abundant lipids overlapped between sum within an MRM list profiled per sample injection and sum within a lipid class. There were 63 lipids unique to sum within an MRM list profiled per sample injection, and 15 were unique to lipid class standardization. Overall, these findings demonstrate that standardization approaches differ by sensitivity and stringency, which can influence the number and type of lipids significantly different between experimental groups.

**Figure 19.**
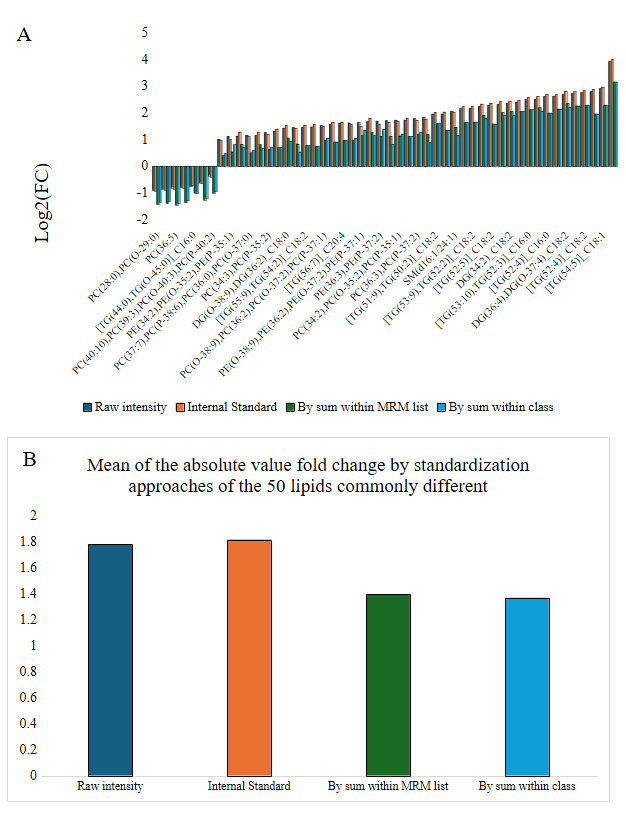
Impact of Standardization Strategy on Magnitude of Differential Lipid Abundance. Clustered bar plot showing the log2 fold change between ZH and SOS for the 50 common lipids to all four standardization approaches. Standardization methods analogous to molar concentration (raw intensity and IS) produced larger magnitudes of log2 fold change than approaches based on molar proportion (sum within MRM list profiled per sample injection and sum within lipid class). (B) Bar plot presenting the mean absolute value log2 fold change between ZH and SOS by standardization approach of the 50 commonly different lipids.

In order to better understand if the standardization approach impacted the magnitude of differential abundance, the log_2_ fold change between ZH and SOS of the 50 lipids common to the approaches were graphed (Figure 19). Standardization strategies analogous to molar concentrations demonstrated larger magnitudes of log_2_ fold change than when standardized as molar proportions. Next, within each family of methods (molar concentration and molar proportion), we compared each standardization method to identify how much the FDR cut-off in the more selective method (standardization by lipid class) would need to be relaxed to retain all lipids with FDR <0.05 in the more sensitive method (standardization by MRM list). For molar proportion methods, the standardization by sum within lipid class FDR needed to be relaxed from 0.05 to 0.98 to include all lipids identified as significant in standardization by sum within the MRM list. Standardization by sum within an MRM list appeared to be much too sensitive in identifying lipid species as different within PE, CE, Cer, PS, PI, and SM classes, as multiple lipids within these classes had FDR >0.25 when data were standardized by sum within lipid class.

**Figure 20.**
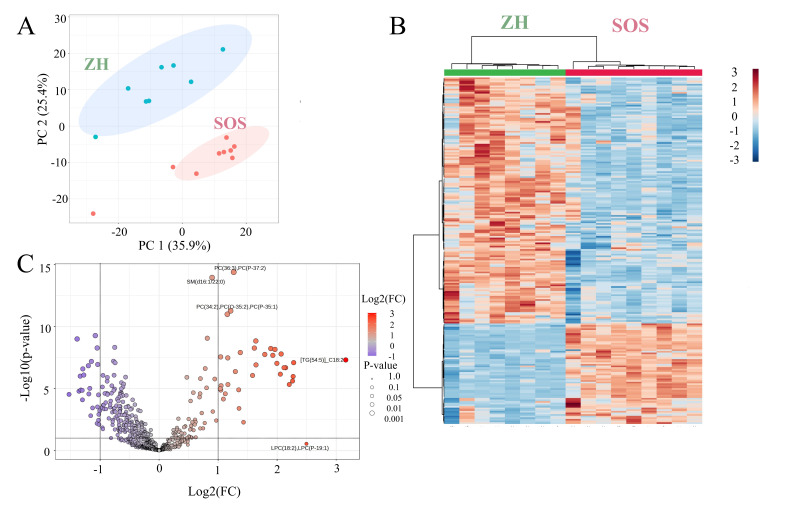
Example of Statistical and Machine-Learning Analyses Performed on Liver Lipids Standardized by Sum Within Lipid Class. K-means clustering (unsupervised machine learning) applied to SOS and ZH samples, showing distinct grouping by treatment. (B) Hierarchical clustering analysis is represented by a dendrogram with a heatmap of standardized lipid intensities, illustrating separation of samples by treatment. (C) Volcano plot displaying log2 fold change (ZH vs. SOS) versus -log10 (raw *p*-value).

Multiple statistical, analytic, and machine leaning approaches were applied to the data standardized by sum within lipid class to demonstrate analyses that can be performed using Metaboanalyst 6.0. For example, K-means clustering, an unsupervised machine learning approach that groups data points based off similarity (Figure 20A), revealed distinct grouping by treatment with tight clustering in SOS and more dispersion in ZH. The spread among ZH is consistent with the increased variability in lipid profiles at birth, likely reflecting biological variation among piglets born to different sows. Hierarchical clustering provided a visual representation of the separation between treatment and how lipids behave in these systems (Figure 20B). A volcano plot creates a visualization of *t*-test and fold change, allowing the fast identification of features that are both substantially and significantly altered between two experimental groups. In other words, a volcano plot can be used to understand the impact of biological conditions on lipid profiles (Figure 20C). These statistical analyses serve as a framework to identify lipids of interest to guide biological interpretation.

**Figure 21.**
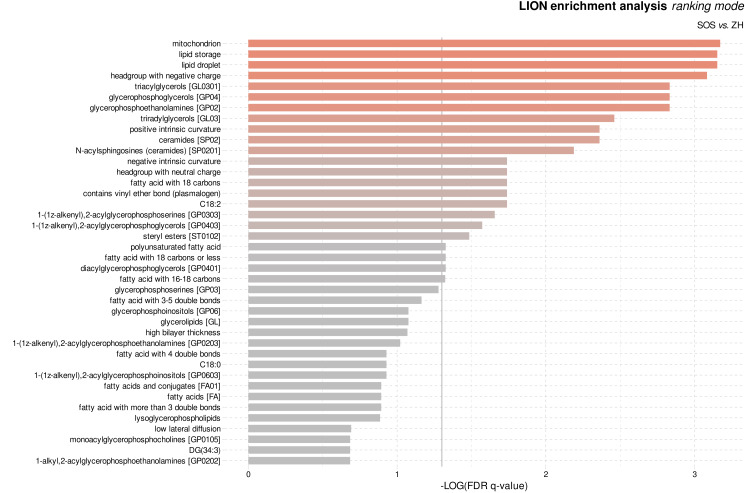
Lipid Ontology-Based Pathway Analysis of Liver Lipids. LION enrichment analysis (ranking mode) comparing SOS to ZH using a one-tailed Kolmogorov-Smirnov test (SOS > ZH). Colored bars indicate terms significantly enriched in SOS.

Data interpretation is the step that transforms statistical outputs into meaningful biological insights. Pathway and enrichment tools such as LION or Metaboanalyst 6.0 have been developed to better enable biological interpretation of lipidomics by providing a complementary perspective by linking changes to cellular and organelle processes.^24^ These enrichment and pathway analysis tools allow lipidomic changes to be interpreted within cellular function categories and organelle association. Using LION, several biological descriptors were enriched in the SOS group compared to ZH (Figure 21). The enrichment analysis identified upregulation of features related to mitochondria, lipid storage, lipid droplet, and headgroups with negative charge. These findings suggest alterations in energy metabolism and membrane composition in response to early postnatal feeding. Although LION was used for this analysis, similar functional interpretations can be obtained in different tools depending on platform preference.

**Figure 22.**
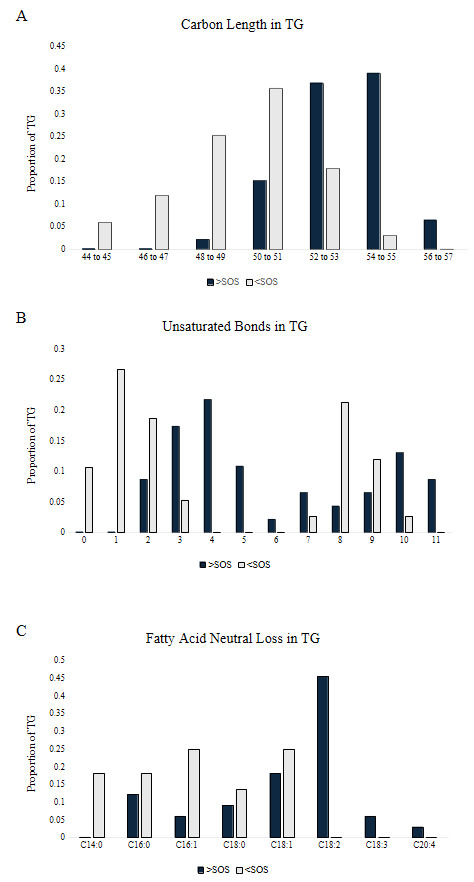
TG Structural Characteristics in Liver of ZH and SOS Piglets. Structural characteristics were derived from TG species standardized by sum within lipid class with FDR <0.05 (*t*-test) by extracting the total carbon number, degree of unsaturation, and fatty acid NL from the lipid names. (A) Proportion of carbon length in TG. (B) Proportion of unsaturated bonds in TG. (C) Proportion of fatty acid NL in TG.

Lipid structural characteristics can reveal patterns in lipid metabolism and storage. This analysis showed that the SOS group had a greater proportion of longer chain TGs (52 to 57), while the ZH group had a greater proportion of shorter chain TGs (44 to 51; Figure 22A). Analysis of the distribution of the total number of unsaturated bonds in TG showed that SOS had a higher proportion of polyunsaturated TG compared to the minimal unsaturated TG in ZH (Figure 22B). The pattern of unsaturated bonds was further supported by the fatty acid composition in TG, where SOS exhibited more polyunsaturated fatty acids like C18:2. Likewise, liver tissue of ZH had a higher proportion of saturated and monounsaturated fatty acids (Figure 22C). Together, these structural analyses demonstrate how evaluating features of lipid classes can uncover patterns in lipid metabolism. Examining structural features such as chain length and unsaturation reflects the patterns in metabolism, membrane remodeling, and lipid storage.^25^ At a broad scale, lipid class distributions can provide an overview on how storage and membrane lipids shift under different conditions.

Application of this workflow to the neonatal piglet liver lipidome demonstrated how data processing enables biological interpretation of MRM profiling results. Our standardization technique (by sum within lipid class) aligned with our biological question to compare compositional changes of the liver lipidome between SOS and ZH piglets. Statistical analyses revealed distinct clustering by treatment, with tighter groupings in SOS than ZH suggesting more heterogeneous lipid profiles at birth, indicating individual dam influence on the neonate.

### Statement of IACUC

The samples from animals analyzed were from a larger study that was reviewed and approved by the Institutional Animal Care and Use Committee of Purdue University (Purdue IACUC Protocol No. 2110002200).

### Ethics Statement

The samples analyzed were from a larger study that was reviewed and approved by the Institutional Animal Care and Use Committee of Purdue University (Purdue IACUC Protocol No. 2110002200).

### Corresponding author

Christina R. Ferreira, Bindley Bioscience Center, Purdue University, 1203 W State St, West Lafayette, IN 47907; Phone: (765) 494-4905; Email: cferrei@purdue.edu;
